# The impact of AI-based painting technology on children’s creative thinking

**DOI:** 10.3389/fpsyg.2025.1598210

**Published:** 2025-10-16

**Authors:** Anna Wang, Yuxin Zhang, Anman Wang, Wei Zheng

**Affiliations:** ^1^Faculty of Education, Jiangxi Science and Technology Normal University, Nanchang, China; ^2^Fengzhuang Primary School, Shouxian, Huainan, China; ^3^College of Fine Arts, Jiangxi Science and Technology Normal University, Nanchang, China

**Keywords:** AI-based painting technology, art education, children’s creative thinking, systematic review, educational technology

## Abstract

With the emerging application of artificial intelligence (AI) in education, AI-based painting technology has been fully introduced into the development of children’s creative thinking. However, the following studies mainly focus on the technological development and system optimization. There are concerns on the limited educational perspectives, sampling biases, and imperfect theoretical frameworks which restrict the effective evaluation on the AI tools and children’s art education. In this paper, we conduct a systematic review and examines the multidimensional impacts of AI-based painting tools on children’s creative thinking, including the creative expression, original creation, independent thinking and problem solving. With the PRISMA guidelines, we analysis 20 empirical articles (14 from Scopus and Web of Science and 6 from other sources) and provide recommendations on how educators can effectively integrate these tools into art education to foster children’s creative thinking. We find that AI tools enhance creative expression through virtual interaction and personalized learning. However, there are still risks of cognitive homogenization due to standardized interfaces and the disconnection between theory and practice. Our study propose evidence-based strategies for educators to select age-appropriate tools and implement process-oriented assessments. We also advocate the application of technology from educational and humanistic perspectives to balance technical effectiveness and humanistic educational goals.

## Introduction

With the development of technology and science, creative thinking, an indispensable skill in the 21st-century (Gu et al., 2019), has been fully recognized to involve and enhance the development of multiple dimensions such as efficient problem-solving (Ali et al., 2021; Pan et al., 2021), independent thinking (Pan et al., 2021), original creation (Al Hashimi et al., 2019; Ali et al., 2021), and creative expression (Berson and Berson, 2024; Gu et al., 2019). The nurturing creative thinking in childhood not only influence the short-run childhood performance but also influence the long-run cognotive and non-cognitive ability (Torrance, 1967; Gu et al., 2019). This is further supported by research indicating that digital technologies play a crucial role in enhancing the quality of early childhood education environments (Hatzigianni et al., 2023).

With the emerging application of AI technology in education (Zhang and Aslan, 2021), research such as that by Agostinelli et al. (2021) on AI as a collaborative learning partner demonstrates its potential role in educational contexts. The following research has found that AI-based painting tools leverage positive effects on the development of children’s creative thinking (Berson and Berson, 2024; Sun et al., 2022). For instance, AI painting serves as a vital medium for creative expression (Murcia et al., 2020; Creely and Blannin, 2025), and generative AI more broadly is increasingly understood as a creative partner in educational contexts (Giannakos et al., 2025). However, the existing literature is predominantly technically oriented (Su and Mokmin, 2024) and focusing on technical development, strategy design, or system optimization with little attention given to educational perspectives (Chen et al., 2020; Zhang and Aslan, 2021), including how assessment should be conceptualized in such innovative learning environments (William, 2022). There is few research on the effects of AI painting on the pedagogical, psychological, and ethical dimensions. Additionally, the following studies mainly highlight the positive effects of AI (Chen et al., 2022; Shen et al., 2022; Ali et al., 2021; Zhang et al., 2022) but overlook the potential adverse effects including cognitive homogenization and over-reliance on technology (Giannakos et al., 2025) which leads to an incomplete understanding of how AI truly influences creative processes. This gap is further compounded by methodological limitations in measuring outcomes, such as ceiling effects that may obscure true impacts (Resch and Isenberg, 2018; Wang et al., 2008).

In this paper, with the PRISMA guidelines and a rigorous review of empirical literature, we systematically analysis the multifaceted effects of AI-based painting technology on children’s creative thinking. Specifically, we examine the mechanisms of how these tools affect creative creative expression, original creation, independent thinking and problem solving which are the main dimensions of the creative thinking. Finally, we also explore pedagogical strategies with the meaningful integration into art education. Therefore, we conduct this study to address the following questions:

1. What is the impact of AI-based painting technology on children’s creative thinking?
2. How can educators effectively integrate AI-based painting tools into art education to foster children’s creative thinking?

Our work primarily contributes to three strands of literature. First, we explore the common knowledge on the educational and humanistic perspectives (Berson and Berson, 2024) and fill the gap between the AI functionality and pedagogical intentionality. Second, we systematically and critically analysis the following fragmented empirical findings (Chen et al., 2022; Shen et al., 2022; Ali et al., 2021; Zhang et al., 2022) and present a view of AI’s dual role in enhancing and potentially inhibiting creativity. The following studies often emphasize positive outcomes with neglecting ethical and cognitive risks. Finally, we propose a process-oriented evaluation framework aligned with developmental appropriateness, moving beyond outcome-focused metrics that dominate current research. The following studies adopt non-random sampling methods, such as convenience sampling (Chen et al., 2022; Su and Yang, 2024; Villegas-Ch et al., 2022), which compromises data representativeness and triggers sampling bias (Nielsen et al., 2017). There are few studies have adopted solely quantitative methods (Shen et al., 2022; Sun et al., 2022), making it difficult to comprehensively explore children’s behaviors.

The remainder of the article is structured into three sections. The first section outlines the methodology and details the inclusion and exclusion criteria used for literature selection. The second section, based on the screened literature, identifies the positive and negative influences of AI-based painting technology on children’s creative thinking. Accordingly, it explores how teachers can select appropriate AI-based painting tools for teaching and effectively evaluate creative thinking. The final section discusses the methodological limitations and outlines potential directions for future research.

## Method

### Types/sources of data

#### Search strategy

**Figure 1:**
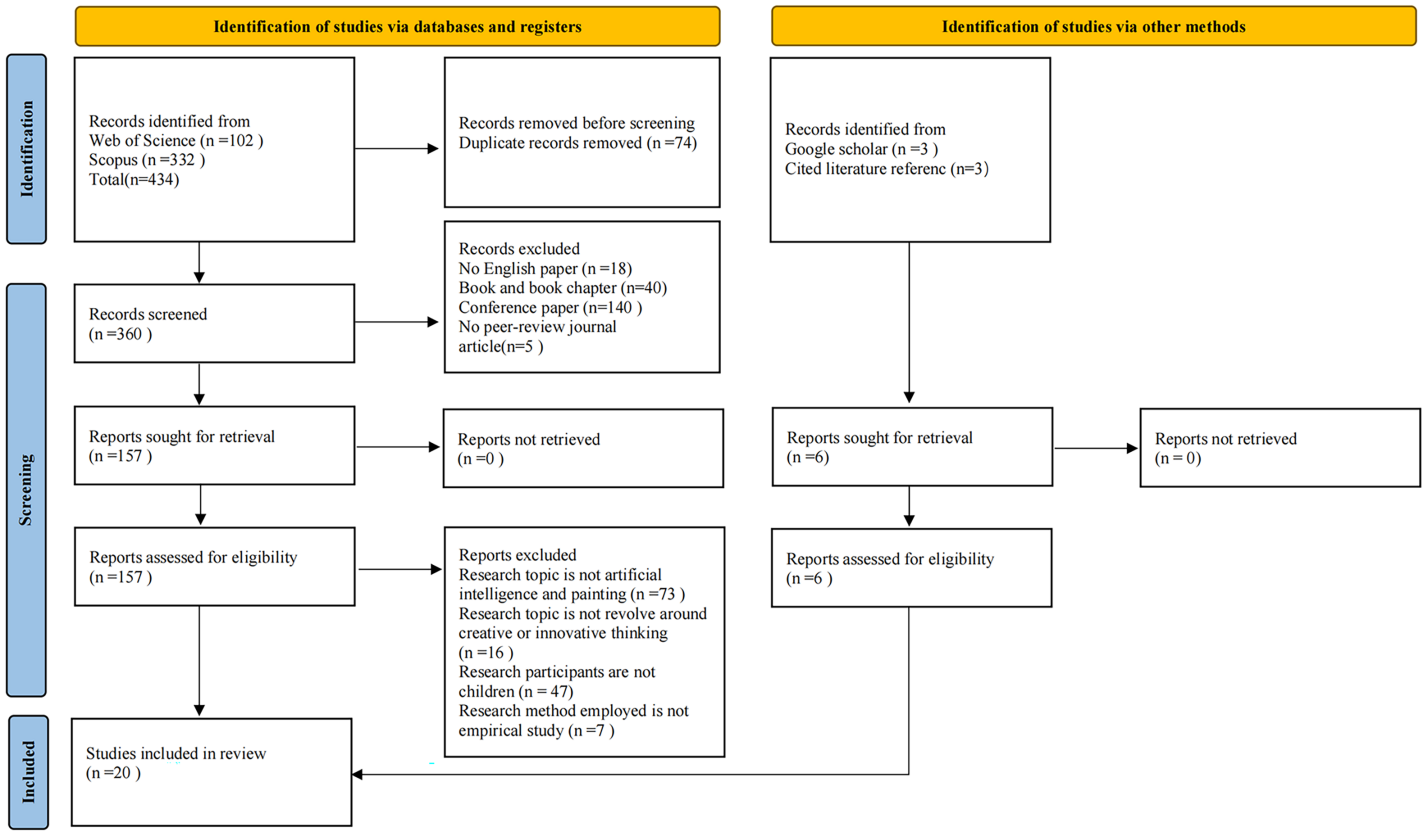
PRISMA flow diagram of literature screening.

The article selection process rigorously followed the Preferred Reporting of Items for Systematic Reviews and Meta-Analyses (PRISMA) 2020 guidelines (Page et al., 2021). The search strategy was designed and reported in accordance with the PRISMA-Search extension recommendation. As illustrated in Figure 1, the process consisted of four main stages: identification, screening, eligibility assessment, and inclusion.

**Figure 2:**
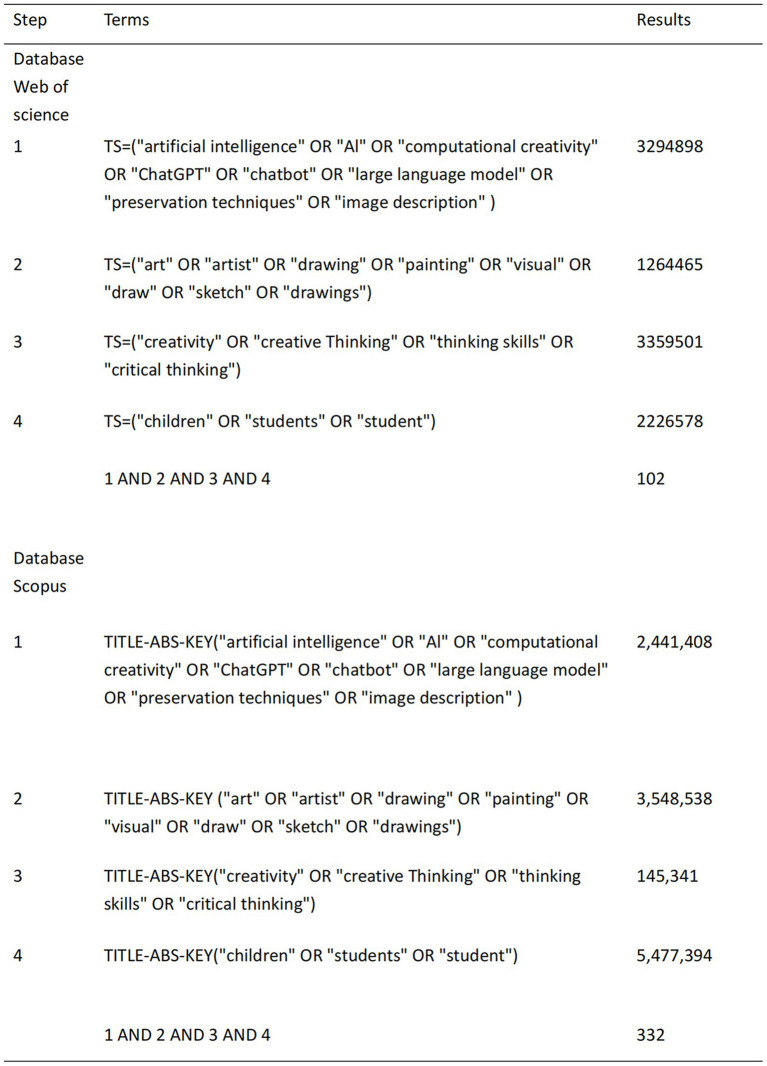
Search strategy in web of science and scopus.

A systematic literature search was conducted on December 5th, 2024, across two major electronic databases: Scopus and Web of Science Core Collection. The search strategy was developed iteratively through pilot searches and by reviewing search strategies used in previous relevant systematic reviews (Ardoin and Bowers, 2020; Oksanen et al., 2023; Samaniego et al., 2024). The final search query combined keywords and index terms related to three core concepts: (1) “artificial intelligence” OR “machine learning” OR “deep learning” OR “computer vision”; (2) “painting” OR “drawing” OR “art creation” OR “visual art”; (3) “child” OR “adolescent” OR “youth” OR “student.” Boolean operators (“OR” within concepts, “AND” between concepts) were applied to refine the search. The complete search syntax for each database, including all field codes and search parameters, is documented in Figure 2. No date or language restrictions were applied during the initial search to maximize retrieval.

All identified records were imported into Zotero reference management software, where duplicate records were removed automatically followed by manual verification, resulting in the exclusion of 74 duplicates. The remaining 360 unique records underwent a two-stage screening process: first based on title and abstract, followed by full-text assessment. Two independent reviewers screened all records according to predetermined eligibility criteria (e.g., empirical studies, focus on AI-assisted painting, involving participants under 18 years old). Any disagreements between reviewers were resolved through discussion or consultation with a third reviewer.

To ensure the consistency and reliability of data extraction, all included studies were coded independently by two reviewers using a standardized data extraction form. The form was pilot-tested on a subset of studies and refined iteratively to improve clarity. The coding procedure encompassed key domains such as study design, participant characteristics, AI intervention details, and outcome measures. Inter-rater reliability was assessed using Cohen’s kappa for categorical variables and intraclass correlation coefficient (ICC) for continuous variables, demonstrating excellent agreement (*κ* = 0.88; ICC = 0.92). Any discrepancies between reviewers were resolved through discussion or by consulting a third reviewer to reach consensus.

Data from included studies were extracted using a standardized form that captured information such as study characteristics, participant demographics, AI intervention details, outcome measures, and key findings. The entire selection process, including reasons for exclusion at the full-text stage, is comprehensively documented in the PRISMA flow diagram (Figure 1).

#### Inclusion and exclusion

We applied a series of inclusion and exclusion criteria. Articles were included if they were: (i) written in English; (ii) published in a peer-reviewed journal; (iii) focused on AI-based painting and creative or innovative thinking; (iv) included children as research participants; (v) employed an empirical research method. Of the 360 records screened, we excluded 180 due to their classification as book chapters or conference papers, and 18 for not being in English, leaving a total of 157 articles for retrieval. We were able to find the full text of all articles, resulting in 157 articles for full-text retrieval. We successfully accessed the full text of all 157 articles. During the full-text review, we excluded 89 articles that did not include AI-based painting and creative thinking, 47 without child participants, and seven that did not adopt an empirical research method. Additionally, through reference list screening and Google Scholar searches, we identified six additional relevant articles. Ultimately, 20 articles met the eligibility criteria and were included in the review sample for data analysis and quality assessment.

## Results

This section presents findings related to the two research questions: (i) compared to traditional painting education models, the integration of AI-based painting technology in children’s art education across multiple dimensions, at varying depths, and through diverse forms of expression; (ii) we examine how teachers can integrate AI into painting classes to enhance children’s creative thinking and explore methods for assessing improvements in their creative thinking.

### Advantages and disadvantages of utilizing AI-based painting technology on children’s creative thinking

#### Enriching children’s creative expression through virtual tools and interaction

AI-based painting technology offers significant advantages for fostering creativity in children, both cognitively and socially. By providing diverse digital materials, tools, and virtual environments, it creates a rich medium that stimulates exploratory and imaginative behaviors beyond the constraints of physical resources (Al Hashimi et al., 2019). This digital affordance is particularly conducive to improvisational and game-based learning, which enhances interpersonal collaboration and shared creative experiences among children (Berson and Berson, 2024).

Furthermore, empirical studies suggest that AI-assisted painting platforms facilitate greater creative output compared to traditional paper-based methods. Children not only choose more varied and unconventional themes (Chen et al., 2024; Kucirkova and Sakr, 2015) but also produce ideas with significantly higher originality and innovative quality (Shen et al., 2022). Such enhancements are often attributed to AI’s ability to lower the threshold for visual expression while raising the ceiling for creative possibility. For example, systems like StoryDrawer leverage abstract sketch interpretation to activate visual thinking and strengthen ideational fluency (Zhang et al., 2022). Similarly, social robotics and AI agents serve as creative partners that children can imitate and learn from, thereby internalizing novel creative strategies through observational learning (Ali et al., 2021).

At a cognitive level, AI tools reduce extraneous load by automating labor-intensive processes such as converting narratives into visuals or handling multi-sensory inputs. This offloading allows children to allocate more cognitive resources toward generative and creative thinking (Zhang et al., 2022). Moreover, emotional and motivational support provided by AI through responsive and adaptive feedback, further engages young learners and encourages creative risk-taking (Shen et al., 2022). Thus, AI does not merely serve as a tool but functions as a cognitive and social scaffold that extends children’s creative capacities.

#### Personalized learning sparks children’s exploratory spirit and independent thinking abilities

AI-based painting technology offers significant potential to enhance learning: (i) AI-based painting technology serves as a dynamic catalyst for children’s artistic cognition and creative autonomy (Rong et al., 2022); (ii) AI-based painting technology not only cultivates personalized expression in digital art (Berson and Berson, 2024; van Breemen et al., 2011) but also facilitates their systematic acquisition of core artistic principles, such as color theory (Chen et al., 2022) and line composition (Shen et al., 2022); and (iii) the dynamic feedback mechanisms in AI systems transform technical acquisition into cognitive exploration processes, where children actively experiment with stylistic variations (Sun et al., 2022) and refine their visual expressions (Berson and Berson, 2024; He and Sun, 2021).

This adaptive learning environment promotes creative risk-taking (Al Hashimi et al., 2019) and enhances problem-solving skills (Rong et al., 2022; Zhang et al., 2022). Real-time interaction with AI tools improves students’ contextual sensitivity to artistic elements (Lim et al., 2023; Rong et al., 2022), stimulating their exploratory thinking and innovative cognition. Through AI systems, children can iteratively experiment with, adjust, and refine their artworks (Shen et al., 2022). This interactive modality not only boosts their confidence in developing unique artistic (Chen et al., 2024) but also advances their meta-cognitive capabilities (Zhang et al., 2022). Collectively, these processes contribute to the progressive evolution of creative cognition (Sun et al., 2022).

#### Challenges of AI-based painting technology in fostering children’s creative thinking

There are potential challenges hindering the development of children’s creative thinking with the application of AI-based painting technology into the learning. First, there are technical and design limitations in the current AI-based painting systems. On one hand, them insufficient personalization leads to homogenized thought processes and curb creative expression (Vartiainen and Tedre, 2023). There is also few tailored support in systems designed for children (Shen et al., 2022). On another hand, AI integration’s effectiveness in improving creativity remains limited while enhancing situational awareness (Lim et al., 2023). The prolonged exposure also foster technological dependency, thereby impairing autonomous thinking in children (Berson and Berson, 2024).

Second, there are systemic and operational issues in the application of AI painting tools for the disrupted children’s creative workflows by the AI architectures. The delayed responses and misinterpretation of instructions may cause frustration and hinder narrative fluency and cognitive engagement during creative tasks (Zhang et al., 2022).

Third, and most critically, the ethical and social dimensions of AI pose profound developmental concerns. The misuses or overuse of AI in painting education may negatively impact children’s physical and mental health, as well as their social skill development, further impeding creative growth. For instance, poorly designed AI feedback mechanisms can internalize negative self-perceptions and inhibit creative risk-taking (Chen et al., 2022). Beyond these impacts, AI applications introduce substantial ethical controversies, including data privacy issues, algorithmic bias, and the potential commercialization of childhood creativity. The opaque nature of AI decision-making raises concerns about whose values are embedded in these systems and how they shape children’s worldviews and creative agency.

Finally, the absence of a robust theoretical framework complicates the design and implementation of AI tools aimed at creativity enhancement. Without a nuanced understanding of the interplay between AI and cognitive development, educational applications risk prioritizing technological innovation over pedagogical integrity and developmental appropriateness (Su and Mokmin, 2024).

### How to effectively integrate AI-based painting tools into art education to foster children’s creative thinking

#### Teachers should select AI-based painting tools suitable for children’s age development characteristics

With the integration of AI-based painting technology into teaching, it is essential for educators to select tools that align with the developmental characteristics of children at different ages. The selected systems should incorporate user-friendly interfaces, simple operations, and adaptability to children’s cognitive abilities. For instance, platforms such as Culture Craft offer intuitive interfaces that require no prior technical knowledge, making them highly accessible for young learners (Berson and Berson, 2024; Sun et al., 2022). Effective tools should also accommodate a range of ages and skill levels to inclusively support digital art creation.

AI painting systems can generally be classified into two types: feedback-based and collaborative (Shen et al., 2022). These tools often utilize devices such as digital drawing tablets (Chen et al., 2024; Shen et al., 2022; Sun et al., 2022), smart glasses (Chen et al., 2022), and robots (Ali et al., 2021; Zhang et al., 2022). Technologically, many systems employ generative adversarial networks (GANs) to generate images from user inputs or predefined styles (Chen et al., 2022; Sun et al., 2022), and support features including style transfer (Lim et al., 2023), imitation (Sun et al., 2022), and collaborative drawing.

Some AI-based painting tools, such as StoryDrawer (Zhang et al., 2022) and ChatScratch (Chen et al., 2024), use voice interaction to convert children’s verbal ideas into images, thereby mitigating limitations in written expression. This approach is particularly suitable for children aged 5–7, as it aligns with their developing linguistic capabilities and reduces interaction barriers. However, exclusive reliance on language may not fully capture children’s creative intent, especially given their still emerging verbal skills. In contrast, for children aged 8–10, systems such as those developed by Chen et al. (2022) which incorporate contour recognition, color matching, and proportional calculations. These tools can more effectively stimulate imagination and support detailed creative expression. These features better match their advancing cognitive and motor skills.

As AI technology continues to evolve, teachers should stay informed about new functionalities and evaluate their suitability based on children’s cognitive development levels. By selecting age-appropriate tools. For example, prioritizing voice interaction for younger children and contour-based or proportional tools for older ones, educators can better foster an environment that enhances creative thinking.

#### Painting education should be supplemented by technology, with children’s interests and subjectivity as the main focus

During integration into children’s painting education, AI tools should be positioned as a medium for exploration, imagination, and understanding the world, rather than an end goal. This approach emphasizes the abilities and initiative children demonstrate while using the tools. Since interest is a key driver of learning, interactive experiences can enhance children’s motivation to learn (Sun et al., 2022) and stimulate collaboration and foster creativity (Ali et al., 2021). For instance, collaborative drawing games like Magic Draw provide open-ended environments where children freely express creativity, significantly boosting their creative thinking (Ali et al., 2021). Compared to traditional methods, AI integration effectively supports personalized learning goals (Berson and Berson, 2024). However, educators should avoid overly prescriptive approaches that overlook children’s interests and exploration (Chen et al., 2024). Instead, they should offer tailored guidance, prioritize personal exploration, and respect children’s subjectivity, ensuring AI tools promote the development of creative thinking.

#### Teachers effectively evaluate the implementation process to assess improvements in children’s creative thinking

Evaluating the impact of AI-based painting tools on children’s creative thinking is crucial in painting education (Sun et al., 2022). While researchers commonly employ qualitative and quantitative methods for data analysis (Berson and Berson, 2024; Chen et al., 2024; Zhang et al., 2022). Methods such as the Creative Thinking Drawing Production test (Ali et al., 2021) may not suit routine classroom use.

Observation offers a practical alternative, allowing teachers to focus on children’s engagement with AI tools, such as their image selection, manipulation, responses to visual stimuli, and overall engagement (Sun et al., 2022). As Berson and Berson (2024) suggest, key indicators such as curiosity, exploratory spirit, and creativity should be prioritized. Encouraging children to share experiences and analyze their artwork for evidence of creative expression also provides valuable insights (Berson and Berson, 2024).

However, challenges such as children’s limited expression and teachers’ varying levels of AI proficiency can hinder evaluation. In response, teachers should enhance their observation and communication skills, deepen their understanding of AI technologies, and refine their evaluation approaches. Such improvements ensure an accurate assessment of AI tools’ effectiveness, supporting the development of children’s creative thinking.

## Conclusion

This study analyzed 20 peer-reviewed empirical research articles to investigate the impact of AI-based painting technologies on children’s creative thinking. The findings revealed that AI-based painting tools significantly enhance fluency, flexibility, and originality (Berson and Berson, 2024; Chen et al., 2022; Zhang et al., 2022). The key advantages include augmented creative expression, personalized learning experiences, and improved exploratory behaviors. However, standardized interfaces in AI-based painting tools may limit children’s creative divergence, potentially leading to homogenized outcomes, For example, AI-generated artworks created by children exhibit a significant degree of similarity.

Currently, we acknowledge the following limitations in this study: (i) the inclusion criteria, limited to English-language peer-reviewed journal articles, may exclude relevant studies in other languages; (ii) the analysis may overlook important theoretical contributions that discuss the impacts of AI on education; (iii) the focus on AI-based painting tools may also fail to capture the wider implications of AI technologies in creative education. Consequently, these findings should be interpreted with caution, considering these limitations. Future research could incorporate databases such as Taylor & Francis Online, SAGE, and Google Scholar, to enhance literature coverage and mitigate selection bias. At the same time, non-English research literature can be included to explore cross-cultural variations in the effects of AI-based painting tools on children’s creative thinking.

This review offers evidence-based strategies for educators to effectively integrate AI-based painting tools while preserving children’s creative autonomy. For instance, it equips educators with insights into aspects such as selecting age-appropriate tools for students, designing personalized learning paths, and measuring creative progress through observational and interactive assessments. These approaches can help address challenges such as technical limitations and cognitive homogenization, to fully unleash the potential of AI in art education.
